# The performance of exceptional public buildings on social media–The case of Depot Boijmans

**DOI:** 10.1371/journal.pone.0282299

**Published:** 2023-02-24

**Authors:** Nadia Alaily-Mattar, Diane Arvanitakis, Hanna Krohberger, Lukas Franz Legner, Alain Thierstein

**Affiliations:** 1 Chair of Urban Development, Department of Architecture, School of Engineering and Design, Technical University of Munich, Munich, Bavaria, Germany; 2 School of Computation, Information and Technology, Technical University of Munich, Garching, Bavaria, Germany; UPSI: Universiti Pendidikan Sultan Idris, MALAYSIA

## Abstract

Exceptional public buildings are buildings that are commissioned by public institutions with the deliberate and declared intention that they become icons. The recognisability of such buildings and their exposure to a wide audience can support the interests of their producers, who are keen on developing symbolic landmarks of their institutions. Textual and visual online communication can play an important role in boosting exposure and affecting how a building acquires iconic status. Content produced by social media users not only reflects how such buildings are perceived, it also goes on to affect how they perform, that is, what narratives they become associated with and how such association supports their transformation into icons. However, the role of content produced by different actors is unclear. In particular, what differences, similarities and influences exist between public/supply and private/user generated social media content particularly during the early life cycle of a building. This article presents a research methodology that can address these questions. Findings generated by applying this methodology on the case study of Depot Boijmans in the City of Rotterdam are presented. By scraping, filtering, organising and analysing content produced by Instagram users about the case study it was possible to show that that public/supply and private/user generated content converge independently. Textual analysis of posts uncovers an overwhelmingly neutral and positive sentiment in posts. Newness, iconicity and the novelty/firstness of the publicly- accessible- art- storage-concept emerge as central topics and are discussed in equal quantities on both the public and private actors. Post behaviour is characteristically different amongst actors, where public actors tell longer stories than private actors but both coinciding with events. The clustering of photographs of the building reveals a trend towards more than one point of interest. The results demonstrate that the exceptional building currently receives more attention on Instagram than the valuable masterpieces stored within it. This suggests the intended performance is achieved in the short term but its long-lasting effects and its assimilation to become an icon in the Museum Park or even the City of Rotterdam will be tested with time. It suggests the photograph itself becomes an actor in the creation of its iconic status in visual media platforms and recognises the agency of non-persons, and that Instagram is merely one of many (social) media platforms used to do so. This research offers methods and their applicability toward a catalogue for data analysis for architecture and urban studies related to the online performance of buildings.

## 1. Introduction

The scope and speed of the digital circulation of photographs of exceptional buildings has increased exponentially with the advent of social media starting in the late 2000’s. The term exceptional buildings is used in this paper to refer to buildings commissioned by actors with the deliberate and declared intention that they stand out amongst the crowd of buildings in cities [1]. These buildings promise to attract significant media attention owing to the high profile status of their architects and the potential recognition value of their designs [2]. Increasingly, social media platforms are the realms within which much of this process of generating and capturing attention unfolds. The textual and visual communication about exceptional buildings by social media users is generating data whose content is large, rapid and unstructured, otherwise known as Big Data. This communicative behaviour of social media users reflects how buildings are received by these users. However, more importantly such communicative behaviour generates content that contributes to a diminishing dominance of supply-led in favour of user-generated content and hence can affect the short- and long-term performance of architecture.

Social media data have remained largely inaccessible to architecture scholars and thus evaded their scrutiny, because architecture scholars lack the necessary skills to collect and analyse such data. In a pilot research project, the authors of this paper, a multi-disciplinary research team composed of scholars from architecture and computational sciences present a research methodology to analyse the transformation of an exceptional building into an icon using social media content scraped from Instagram. In particular using manual analysis of high interaction users, we differentiate between public and private actor groups. A key limitation of previous relevant research is that the profile of social media users is not sufficiently investigated and that users are treated as a homogeneous entity. Findings generated by applying this methodology on the case study of Depot Boijmans in the City of Rotterdam, Netherlands (referred to as the Depot hereafter) are presented. As a collaboration between Museum Boijmans Van Beuningen, Municipality Rotterdam and the De Verre Bergen foundation, the Depot was commissioned to architecture firm MVRDV with the specific request for “an imposing, spectacular and inviting building, which lies like an icon in the Museum Park and attracts attention from afar” [3, translation by authors]. The Depot’s eventual assimilation as an icon in the Museum Park or even the City of Rotterdam can only be tested with time. Analysing the early stage in a building’s life cycle and selected time frame of the research has obvious limitations. Findings may be skewed by, for example, the expected positive reception of the novelty and firstness, notwithstanding the general positivity of Instagram. The decision to follow the “live” unfolding rather than prove or disprove a historical trajectory limited the researchers bias which would need mitigation where knowledge of a given trajectory exists. The open-ended effect of findings enables us to focus more objectively on describing the performance of this building.

The data collected included digital photographs and their associated meta-data, namely account holder, upload date, comments, caption and hashtags based on an automated search for hashtags and the location related to the Depot. In a subsequent step this data was cleaned and organised to generate a dataset. After manually inspecting selected user profiles identified six user groups were identified, which were then classified as representative of the public or private actors and then were examined with respect to the frequency of posts. Through topic modelling, analysis of adjectives and sentiment analysis, the topics discussed and attitudes towards the Depot by users were investigated. By clustering the images in the dataset, important points of interest and their relative popularity within the Depot among the visitors were identified. The goal was to untangle user generated content from supply generated content, identify what textual and visual content is pushed into the social media by different actors, assess the dominance of content generated by different profiles of users and find out whether, and if so how, this content relates to one another. The assumption was that narratives actively pushed by public actors in the social media are amplified by private actors. This paper adheres to Barthes and Duisit [4] definition of narratives as sense-making depictions that influence the way in which an audience perceives a reality presented by a narrator; a narrative transports emotions and value.

Describing how at this early stage in the building’s life cycle the selected case study performs on a social media platform, such as Instagram is significant, because it provides empirical evidence about how the textual and visual communication by different profiles of social media users unfolds and how dominant private actors are in this communication. Such evidence can be used in a later stage to assess the online performance of such a building. In addition, research of other media of communication such as newspapers, press releases, presentations, promotional videos, websites or other social media platforms can provide insights into how content might migrate to exert influence beyond the confines of the initial medium through which it was released. “Ground-truthing findings through site visits and interviews” [5] can enhance the validity of drawn conclusions. Such findings would allow describing the level of control that public authorities have over narratives associated with such buildings. This in turn would support evaluating how a city has steered its urban development.

## 2. Architecture and social media

Exceptional buildings are often commissioned to become icons that affect how their host institutions and even cities are perceived. Often there is a sense of urgency to proclaim that a building has achieved its purpose and indeed become an icon. However, history attests that the transformation of buildings into icons takes time to unfold, time that often exceeds the regular lifecycle of buildings. In addition, iconicity is not a stable or objective condition, rather it can fluctuate with time. It is useful to differentiate between the term “icon” used as a noun to describe a condition, for example, a building is an icon, versus as an adjective to describe physical characteristics, for example, an iconic building or iconic architecture. Used as a noun, iconicity refers to the condition of being representative or symbolic of something. Used as an adjective, it refers to an unconventional architecture which according to Jencks [6] use “enigmatic signifiers” and “carries significant and relevant suggestion”. Iconic architecture does not necessarily transform buildings into icons, nor do all buildings that have become icons have necessarily an iconic architecture. As such iconic status is related to meaning that “comes not from aesthetic surface but from society, from somewhere outside the objects themselves” [7]. Understood as such, the assumption is then that communication via social media can accelerate the transformation of buildings into icons. Sklair [8] notes that the circulation of photographs is central in the production and iteration of iconicity. The photographs become representative of lifestyles, meanings and values that do not necessarily relate to the programme of the building itself [9]. Users’ sharing of digital photographs and text related to an exceptional building captures data not only about a user’s “[expectation] to be transformed by the experience, that it will leave us seeing the world a little differently” [10], but also about how iconicity may or may not unfold. Data hosted on social and photo-sharing media platforms present a great potential for uncovering how exceptional buildings are perceived and how communication about this perception goes on to change the way these buildings perform.

In the social sciences, the rush to use data of social media platforms resulted in a "data gold rush" [11] and a “computational turn” [12]. In the spatial sciences there is an equal appeal for using such data for describing, measuring and understanding spatially relevant phenomena. Table 1 provides an overview of the focus and limitations of some of the main recent publications relevant to the study of iconicity and architecture. Analysing the city through data retrieved from Location Based Social Networks (LBSNs) has received considerable attention as a promising method for applied research [13]. Currid and Williams [14] use geotagged photographs that help identify macro-geographical patterns. Similarly, using georeferenced social media data to map the sensorial and emotional layers of cities researchers offer an alternative cartographic representation of a city (see for example Quercia, Schifanella [15], and crowd-sourced cognitive map [16, 17] and methods to quantify urban perceptions [18]. Using data scraped from social media has been increasingly popular by scholars studying landscape characterisation and perception [19]. “Analysis of geolocated photographs is a simple and fast way to evaluate the aesthetic value of the landscape and its appreciation by people” [20]. However, Callau et al. [21] point to the need to address the limitations posed by the heterogeneity of social media users. They scrape photographs from Wikiloc, a crowdsourced sports platform for outdoor activities, whose users are more homogeneous. Nevertheless, they conclude “Although contents of the photographs can be clearly tagged, their meaning is difficult to interpret because they mainly respond to visitor experiences” [21]. Song and Zhang [22] address this limitation by correlating the image data with the hashtag data as a way of making more sense of the data. Similarly, Geboers and Van De Wiele [23] correlate the visual elements of photographs with their associated hashtags to add a contextual dimension into the analysis of images.

**Table 1:** Overview of recent research relevant to the study of iconicity and architecture.

	Authors	Focus of study	Limitations and differences ^(^*^)^	Case Study	Data Source
Theory	Jencks (2006, 2011) Alexander (2012) Sklair (2017)	Theoretical understanding of iconicity and architecture.	Lack of empirical evidence. No consideration of the role of social media.	none	none
	van Dijck (2008) Serafinelli (2018)	Theoretical understanding of digital photography.	Lack of empirical application.	none	none
Media studies	Geboers et al. (2020)	Correlating the visual elements of photographs with their associated hashtags to add a contextual dimension into the analysis of images.	Focus is on an event and not on built environment. Lack of analysis of the profile of users.	An event, the chemical attack in Aleppo, Syria	Twitter
Tourism studies	Marine-Roig and Clavé (2016)	Analyzing the destination image of a region using metadata (post frequency) and content analysis (counting the most frequent words) with relation to cognitive and affective component.	Data mining process requiring substantial informatics knowledge. Content analysis is limited to keywords, more in-depth methods for topic analysis and sentiment analysis are possible.	Catalonia, Spain	Reviews on Travel-review websites including TripAdvisor.com, TravelBlog.org obtained through web crawling
	Marine-Roig and Ferrer-Rosell (2018)	Analyzing and measuring the (in)congruity or gap between the supply-side projected vs. demand-side perceived Tourist Destination Image.	Automatic analysis of feelings or sensations is based on a feelings lexicon of limited (25) words. Methods that allow keywords to emerge from the content is recommended. Highlights that each platform has its own biases that need to be stated. Limited range of information sources.	Catalonia, Spain	Catalan Tourist Board dossier, Lonely Planet travel guide, and random sample of online travel reviews by tourists who visited Catalonia during 2015.
	Iglesias-Sánchez et al. (2020)	Understanding the impact of Instagram as a co-creation space for tourist destination image building, and as an active component in the destination’s image.	Qualitative analysis, with limited sample size, through content analysis and comparative metrics based on hashtags. Further analysis on institution and user projection of city’s image is suggested. General positivity of Instagram platform needing comparison with other social media platforms is noted.	Algarve (Portugal and Costa del Sol (Spain)	Instagram
	D Paül i Agustí (2021)	Identification of tourist images, manual mapping of the image location through image content. Mapping spatial differences in posts between user-generated content and content posted by tourist boards.	Only spatial analysis. Highlights that different sources should be considered when analysing the tourist image of a destination.	Barcelona, Spain	Instagram
	Egger et al. (2022)	Identifying differences between projected and perceived image of a place using image content analysis; analysis includes image labeling via Cloud Vision, then clustering of labels to produce topic clusters and manual annotation of clusters.	Limited sample size for public side. Only analysis of images, textual content (captions and comments) not analyzed.	Saalbach-Hinterglemm, Austria	Instagram
Spatial analysis and urban studies	Currid and Williams [14]	Identifying macro-geographical patterns, the “Geography of Buzz” using geotagged photographs; the geographic aggregation of events is considered as proxy for social milieu.	The collected digital images do not have metadata, digital images are used as point data only. Investigating content of the images is beyond the scope of the study.	Los Angeles and New York, USA	Getty images
	Salesses et al. (2013)	Measuring the perception of safety, class and uniqueness of places in cities based on assessments of geo-tagged images of these places by survey participants.	Urban perceptions are not extracted from the collected data, but assessed based on an additionally conducted survey.	New York, Boston (USA) and Linz, Salzburg (Austria)	Google Street View for US cities, manual collection on site for Austrian cities
	Quercia et al. (2014)	Quantifying the extent to which urban locations are pleasant using data from a crowd-sourcing platform that shows street scenes and users’ votes.	Metadata is not included in analysis. Users are treated as sensors that offer georeferenced data with which sensorial and emotional layers of cities can be mapped thus offering alternative cartographic representation of a city.	Selected streets in London, UK and Boston USA	Flickr and Google Street views.
	[16, 17]	City cognitive mapping through geo-tagged photos thus showing how the use of computational approach can complement qualitative research.	Digital application of an analogue analysis of the perception of cities using geo-tags, images and hashtags of crowd-sourced data. Methods for image recognition training, clustering, and identification of spatial and architectural elements in a city.	26 cities in Europe, Asia, and North America	Panoramio and Flickr
	Lieskovský et al. (2017)	Evaluating aesthetic values appreciation of different landscape types based on geolocated photographs.	Aesthetic value is correlated to volume of images only.	Slovakia	Panoramio
	Kaußen (2018)	Making the case for landscape characterisation and perception using analysis of social media data.	Methodology is only presented; it is not applied on a case study.	none	none
	Jin and Kim (2018)	Identifying place identity of a city using crowd-sourced text data.	Focus is only hashtag analysis.	four case study cities in metropolitan areas of Seoul, Korea	Instagram (using picodash for scraping)
	Zasina (2018)	Describing the nature of Instagram image of the city by manually classifying the subjects and qualities of the architecture and public spaces featured on images uploaded by users on Instagram.	Manual classification of images. No longitudinal analysis. No analysis of meta data.	Lodz, Poland	Instagram
	Marti et al. (2019)	Reviewing five social networks (Google Places, Foursquare, Twitter, Instagram and Airbnb), highlighting their opportunities and challenges regarding data collection and quality for the purpose of urban studies.	Just a review, not applied research.	none	none
	Callau et al. (2019)	Providing information regarding what draws visitors’ attention to a landscape using image recognition software for tagging and classification of photographs.	Popularity and positive reception of the landscape are correlated to the volume of images uploaded by users of a specific landscape. Authors point to the limitation of interpreting the meaning of tagged photographs, because tags mainly respond to visitor experiences.	Ebro Delta Natural Park, Spain	Wikiloc (crowdsourced sports platform for outdoor activities, users are more homogeneous as opposed to Flickr or Instagram)
	Song and Zhang (2020)	Correlating image data with hashtag data as a way of making more sense of the data.	Particular profile of user groups for each social media platforms is not representative. Similar scenes captured in images makes for homogenous representation of the subject landscape.	Seattle Freeway Park, USA	Instagram

(*) The focus of none of the papers is individual buildings, rather the analysis pertains to streets, city quarters, cities, landscapes, regions or events.

What such studies have in common is the idea that “everyone in a city is somehow transformed to a ‘‘sensor” through his mobile client, receiving and sharing information all the time” [16]. In addition such studies suggest that the crowdsourcing of data allows the interpretation of collective perceptions thereby solving the subjectivity problem in conventional research projects [24]. However, this is problematic in two ways. First, user generated content or what Bernabeu-Bautista, Serrano-Estrada [25] call “volunteered generated information (VGI)” is affected by the platform on which it is hosted. For example, as Zasina [26] argues, Instagram content does not reflect the urban space in general. It rather selects geographies and subjects presenting aestheticized and picturesque places and objects–ratifying it as the go-to platform for photogenic buildings. “Instagram constitutes a distinctive way of seeing that composes an image of the city that is sanitised and nearly devoid of negativity” [27]. In other words, although everyone in a city might be transformed to a sensor, enabling the formation of big data, such data especially if scraped from social media platforms is not objective, rather it can be described as collectively subjective.

The second and more important problem related to the analysis of crowd-sourced data in the spatial sciences is that such analysis often focuses on what information such data captures rather than what it potentially does. In other words, it neglects the agency of both users and data. By sharing data which they both receive and produce [25] social media users affect the performance of buildings, they co-create meaning and symbolism of buildings and institutions and according to Iglesias-Sánchez, Correia [28] even a city’s image or brand. Tourism studies acknowledge that social media should be considered not only as a communication tool, but also as an active component in the destination’s image [28]. The tourist destination image (TDI) has been conceptualized as consisting of two parts, the supply-side (projected) and demand-side (perceived) image [29]. By leveraging methods to obtain and analyse crowd-sourced data [30], the projected and perceived image have been compared [31]. While the two sides differ in certain aspects, like spatial distribution [32], they often overlap in content and can influence each other. “Destinations seize content generated by tourists; therefore, the hermeneutic circle of representation is inverted as photographs taken by tourists aim to reproduce the perceived image of a destination and motivate tourists to capture their experiences with the best picture” [28].

In architecture, the renowned architecture firm Office for Metropolitan Architecture (OMA) has replicated this approach by hosting photographs of their projects that have been taken by users rather than by professional photographers and uploading them on the firm’s website. Initially, under the disguise of post occupancy assessment in 2015, OMA/AMO launched the hashtag #omapostoccupancy, making Instagram a primary tool in its process of gathering understanding of how people use and appropriate buildings. In the words of Giacomo Ardesio, architect at OMA/AMO, “the more a building is capable of engaging somehow the visitors beyond the programme that it is meant to solve, at least from a certain point of view, the more it is successful today” [33]. This approach of architects towards social media has instigated a debate in architecture regarding how the circulation of digital content has become a major concern in architecture practice setting a focus on visuality in architecture and the production of “Instagrammable architecture”.

In order to investigate the performance of architecture on social media, it is important to acknowledge the changed role and function of photography as it has become digital. “[Digital photography] is not about taking photographs but about sharing them. This change means that it has become a tool for an individual’s identity formation and communication” [34]. As van Dijck puts it, young people “take less interest in sharing photographs as *objects* than as sharing them as *experiences*” [35]. A key characteristic of data, including photographs, that is being shared is its potential ubiquity without needing to resort to reproduction. Serafinelli [36] argues that “Benjamin’s concept of reproducibility is substituted by the potential virality and connectivity of the Internet, which play a crucial role in shaping the information sharing” [36].

Acknowledging the collective subjectivity of user generated content in social media and the agency of users opens the field for interrogating the agency of data comprised of digital photographs and text which “is no longer representing reality but helping to form one that sometimes hides more than it reveals” [37]. Social media users share digital photographs and text which co-create this reality. In order to describe this co-creation process it is necessary to collect, clean, sort and analyse social media data. To address the emerging communicative role of photographs and its potential for co-creation of architecture the research question focused on how an exceptional building performs on a social media platform. Performance on social media pertains to the depictions that emerge owing to the quality, quantity and speed of textual and visual communication of different profiles of users. The assumptions were that public/supply side actors push certain sense making depictions i.e. narratives whose purpose is to aid the building’s acquisition of iconic status and that these narratives are picked up, repeated and amplified by users. Hence, to understand this performance it is necessary to find out what differences, similarities and influences are there between public/supply and private/user generated content.

Instagram was chosen as the platform to undertake this research about the selected case study, the Depot, because Instagram provides an environment for users to communicate their experiences both through predominantly photographs and in text form. In contrast, users on comparable platforms like Twitter and Flickr tend to either communicate predominantly in text (Twitter) or be more concerned with sharing photographs as a way of documenting an experience rather than communicating it (Flickr). Additionally, existing research suggests that Instagram more accurately represents visitors’ opinions on a certain place than either Flickr or Twitter platforms [38]. Nevertheless, limitations persist pertaining to the “Instagram positivity bubble” created by cognitive biases [39], self-presentation norms [40], social norms [41], toxic positivity [42, 43], expressing creativity [44] and the inspirational [45] aspects characteristic of the platform.

## 3. The case study: Depot Boijmans

With its extraordinary design, high profile actors and public function, the Depot is a suitable case study to investigate the performance of architecture on social media. Its recent opening in November 2021 during the Corona pandemic provides a unique opportunity to research a live unfolding of this performance amidst little content generated by tourists. The Depot is a publicly accessible art storage facility, designed by the renowned Dutch architecture firm MVRDV. The total cost of 92,5 Mio Euros [46] was financed by a Public-Private Partnership. The inception of the idea of a Depot dates back to the early 2000s. The growing art collection of Museum Boijmans van Beuningen was at risk of being damaged by recurring floods when Sjarel Ex became director of the Museum in 2004. This risk posed an urgent unsolved problem [47] and built the base for conversations about renewing the existing depots in 2005 [48]. From the beginning the plan was to develop something new- a collection building—characterised by iconic and attention drawing architecture, a partly public art storage set to showcase the importance of art as a valuable part of national inheritance [3].

**Fig 1:**
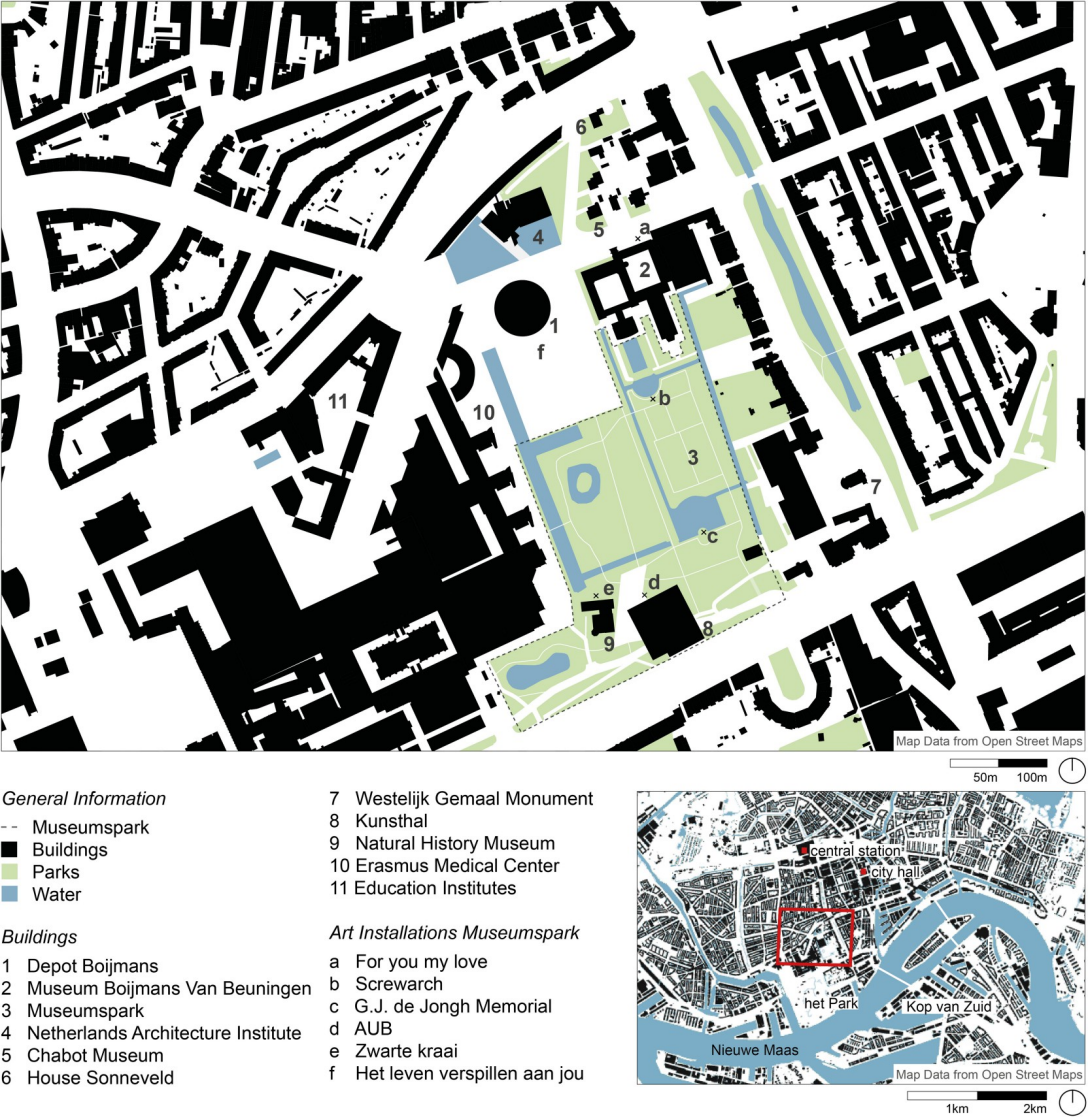
Localisation and context of the Depot Boijmans (by authors).

In 2007 MVRDV first was commissioned to plan a new depot as a “public treasury” for the city next to the Museum [49]. The project was cancelled due to a lack of finances [49, 50]. In 2009 a second attempt to launch the Depot project was initiated. At this time the City of Rotterdam, the Museum Boijmans Van Beuningen, and the Foundation De Verre Bergen formed a Public-Private Partnership brought financial support the project [3]. Based on studies analysing location and feasibility cases, the Museum Boijmans van Beuningen, private service providers and the City of Rotterdam developed the overall requirement profile “Programma van Eisen Collectiegebouw Museum Boijmans Van Beuningen’’ [48]. The site for the Depot is located next to the Museum in the Museumpark, surrounded by other museums, institutions and landmarks *(Fig 1);* the location was chosen in order to create positive synergies [3]. To find a fitting design an Open International Architecture Competition with 47 participants was curated [51]. MVRDV was announced the winner of the competition and construction began in Spring 2017. Following the escalation of costs during construction, the rental of storage space was offered to private collectors which provided sustainable financial viability for the project. After the silver opening in August 2021, the Depot held an official opening on 5th November 2021, attended by King Willem Alexander of Netherlands.

**Fig 2:**
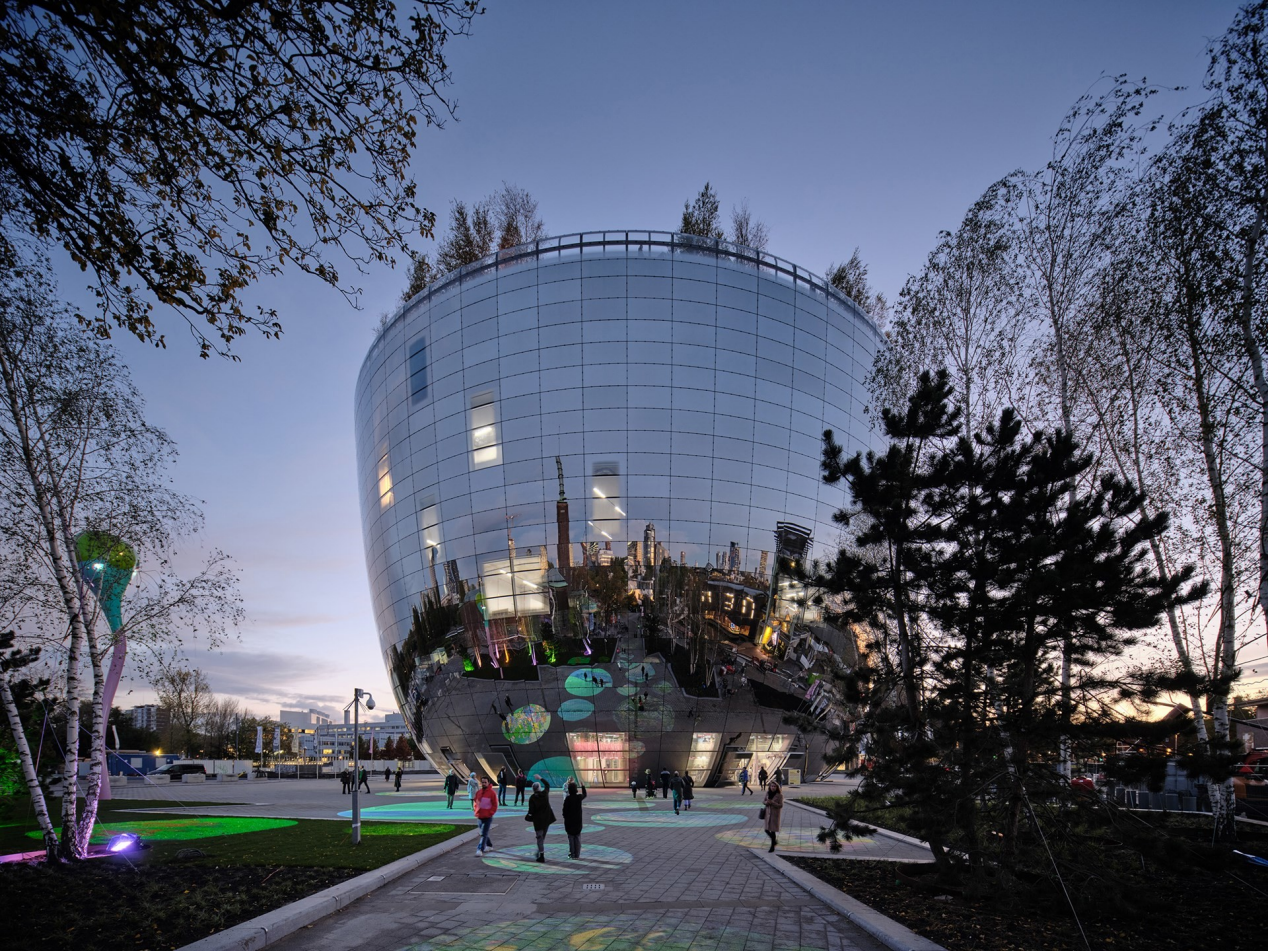
Visualisation of the exterior view of the Depot Boijmans Van Beuningen (MVRDV ©Ossip van Duivenbode).

The Depot consists of six stories of needs-oriented storage space for public and private collectors. To prevent flood damage no art is stored on the ground floor. The distinct form of the Depot reminds spectators of a giant mirrored bowl with a flat forested roof terrace *(Fig 2)*. The plaza reflecting facade and the outside plaza area are used as a canvas to the art installation by Pipilotti Rist. The art collaborator for the lobby was the artist and architect John Körmeling and for the atrium was artist Marijke van Diemen. A prominent interior feature is the Maze: “a three-dimensional labyrinth with floating display cases in which artworks and objects from the storage facilities are placed” [52]. The rooftop terrace and restaurant are designed by Concrete Amsterdam and showcases a view of the City of Rotterdam skyline.

During the daytime the Depot is open for visitors. After buying a ticket in the lobby, visitors can experience the methods of art storage and conservation by following a curated path through the Depot or joining a guided tour. The path reveals about 20% of the building and gives glimpses of the closed off areas through glass panels [3]. After the closing of the Depot in the evening, the restaurant and roof terrace are freely accessible via express elevator. Since its opening, visiting hours have been repeatedly restricted in line with the Dutch government regulations to control the spread of the Coronavirus. Under the conditions of the pandemic, it is reasonable to assume that the posts are dominantly from a local perspective with unusually limited tourist users—a unique local perspective is reflected in this research.

**Fig 3:**
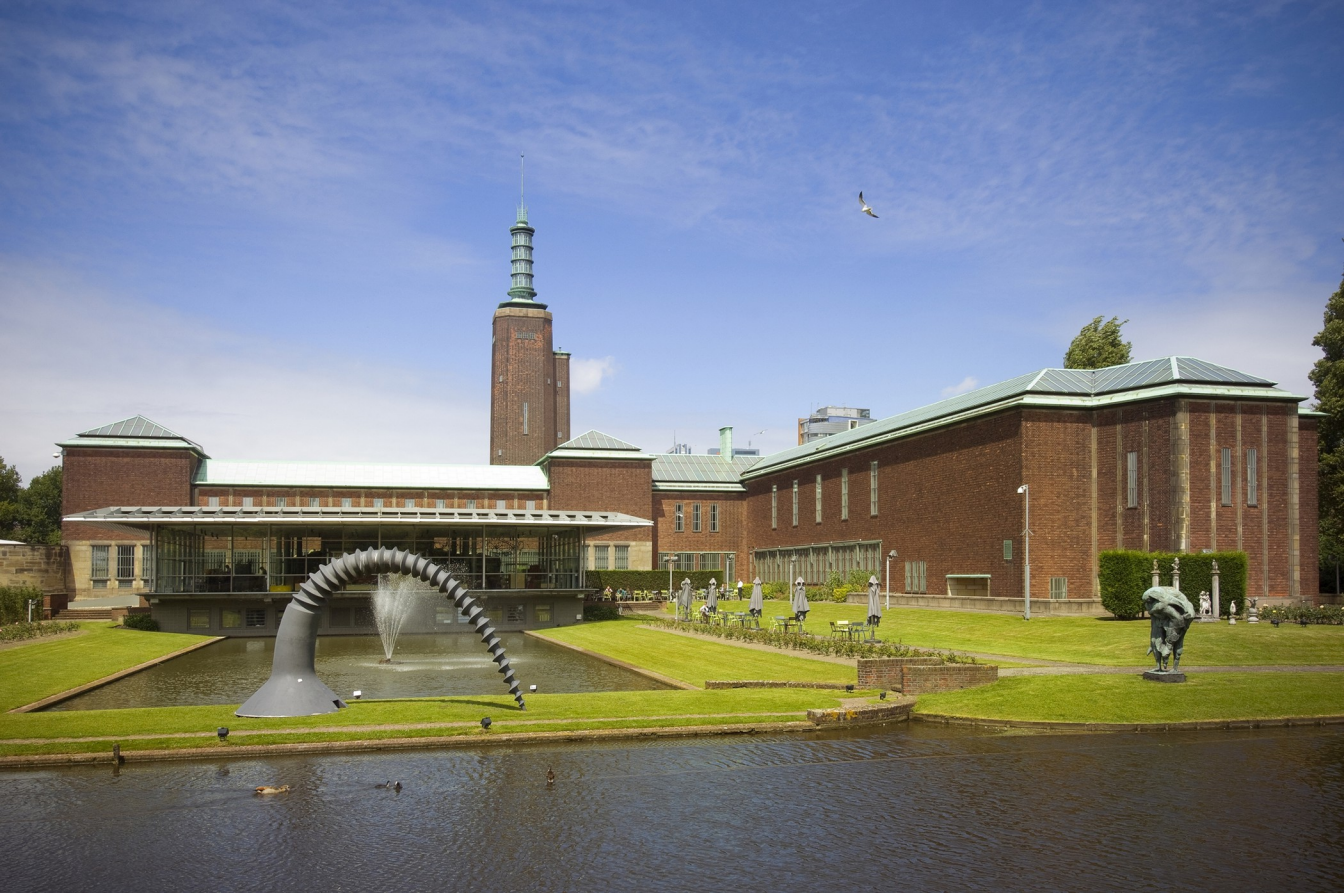
Exterior view of the Museum Boijmans Van Beuningen (studio Hans Wilschut 2008).

It is worth noting that the Museum Boijmans Van Beuningen (*Fig 3*) is currently undergoing renovation and modernization which includes the addition of a new wing by Mecanoo architects. The museum has been closed to the public since 2019 and is expected to open again in 2026 [53].

## 4. Data and methods

Research did not include human participants and therefore consents are not applicable. Personal data from third party sources is used, but the dataset has been anonymised.

### All data sources are acknowledged clearly in this and the following section

Creating the dataset of photographs and captions via scraping by hashtag and location

Photographs on Instagram can be related to a particular topic via two attributes: a location and hashtags. Locations on Instagram correspond to a fixed landmark, such as ’Depot Boijmans van Beuningen’ or ’Rotterdam’. To set a location for their post, a user can select one proposed by Instagram based on their GPS location at the time of posting. Alternatively, the user can search and select from a list of locations provided by the platform. In that sense, the discrete locations used on Instagram are different from locations used on platforms such as Twitter and Flickr, where users can freely choose locations by specifying a longitude and latitude value. All posts relating to the location ’Depot Boijmans van Beuningen’ were scraped for this study.

Hashtags are a way for users to add context to their photographs and make their posts discoverable by other users. Consequently, a set of hashtags were used to search for posts of the Depot on Instagram. The initial set of hashtags contained the two hashtags #boijmans and #depotboijmansvanbeuningen. This set was extended by using a two-step iterative process. First, Instagram’s hashtag discovery feature was used to return related hashtags. Second, the posts yielded by a hashtag search with the current set of hashtags and extracted popular hashtags not yet in the set were analysed. This process was repeated until no new hashtags could be added. In total, 13 hashtags were used to scrape the Instagram for the case study (#boijmans, #depotboijmansvanbeuningen, #boijmansdepot, #boijmansmuseum, #depotboijmans, #boijmansvanbeuningen, #depotrotterdam, #boijmansvanbeuningenmuseum, #boymansdepot, #museumpark, #museumparkrotterdam, #boymansvanbeuningen, #boymans). It is important to note that specific hashtags are related to both the Depot and the Museum Boijmans. Therefore, posts about the museum were filtered out by only considering posts that contained the word ’depot’ in the post description. The dataset was scraped from Instagram using a third-party service provided by RapidAPI (rapidapi.com). Table 2 shows an overview of the dataset (*S1 Data*).

**Table 2:** Overview of the Depot Boijmans dataset (January 2020—December 2021).

Description	Number
Posts	8,228
Users	5,029
Photographs	18,570
Likes	746,742
Comments	32,512

### Identifying public and private actor groups by manual research of high interaction users

In order to understand whether and, if so, how content propagated from the supply/public side actors is received by the demand/private side actors it is important to. differentiate between public actors and private actors. The public actors include: public institutions such as the City of Rotterdam, Museum Boijmans, Rotterdam Citizen Administration, Yoeri Meessen, and Rotterdam Public Administration; designers commissioned for the Depot such as MVRDV (architect), Jan Knikker (employee of MVRDV), Concrete Amsterdam (rooftop café restaurant interior architect), Marijke van Dieman (art collaborator for atrium); and affiliated enterprises who collaborated with the Depot such as Susan Bijl (textile designer) who produced a silver bag to commemorate the silver opening of the Depot. The private actors include: private tourism outlets; enterprises such as photographers, media outlets and fashion promoters etcetera; and individuals.

To identify these actors within the dataset high-interaction users were interrogated. High-interaction users are defined as users with at least five posts about the Depot Boijmans (meaning they show a certain interest in the Depot); and at least 500 followers following the threshold for ‘micro-influencers’ as defined by Rakoczy, Bouzeghoub [54] and Gretzel [55]. 108 such high interaction users were identified in the data set. These make up 2.1% of users and 42.7% of total interactions in the dataset.

**Fig 4:**
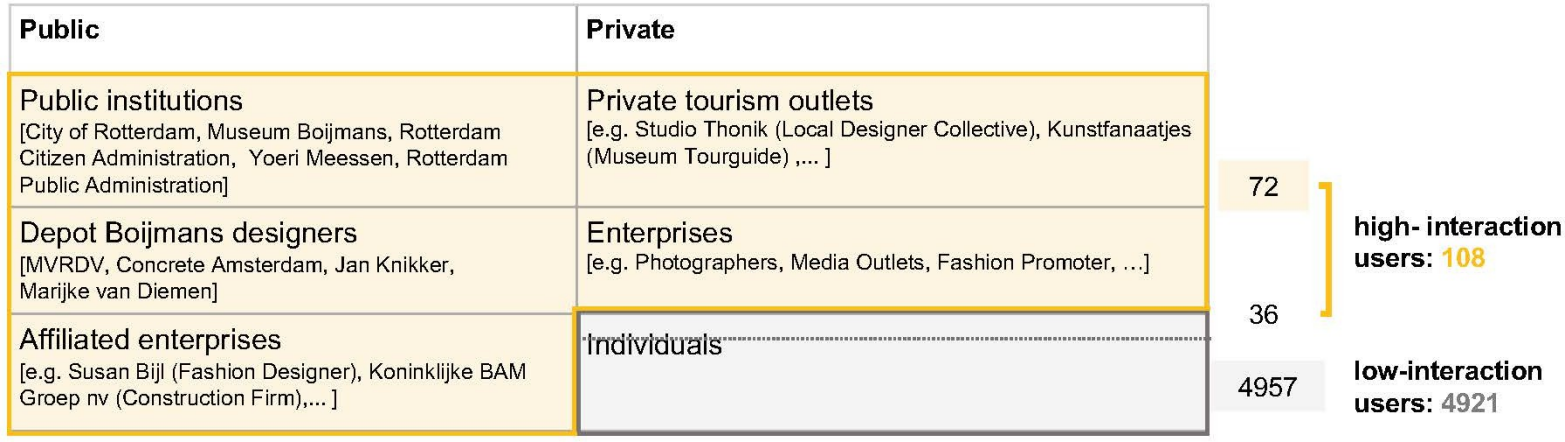
Actors posting on the Depot on Instagram.

These restrictions ensure that the resulting six groups of actors considered in the evaluation have contributed to the media presence of the Depot. Of the 108 high-interaction users, 36 were considered to have no professional relation to the Depot and together with the 4921 low-interaction users make up the group of ’Individuals’. The remaining 72 high-interaction users were labelled according to the five remaining groups (*Fig 4*).

### Identifying points of interest via photograph clustering and labelling

Through an analysis of photograph content, points of interest (POIs) were identified and investigated with regards to their popularity among the posted photographs. POIs were identified as groups of photographs that depicted the same object and thus had similar photographic content. The photographs of the Depot exhibit several sub-spaces, each can be considered a POI for visitors.

In related research, photograph clustering has been used to identify the collective image of a destination based on social media content [31, 56, 57]. Clustering is commonly performed indirectly, through textual image labels given by the Cloud Vision API, which has been shown to produce inconsistent labels when noise is present in the photograph [58]. Therefore, the aim was to cluster photographs directly through their contents, borrowing concepts and methods from image retrieval research [59, 60]. In image retrieval, images are represented by image descriptors which encompass main visual features in the photograph. The problem of finding similar photographs can then be expressed as finding photographs with similar descriptors. Using a convolutional neural network (CNN) [59] the image descriptors were computed. Additionally, *clustering* algorithms provide a way to organise an unstructured dataset into groups of similar data points. A clustering of the image descriptors then yielded candidate clusters for potential POIs. Since the number of POIs within the dataset was initially unknown, a clustering algorithm was chosen that makes no assumptions about the number of clusters ahead of time. Therefore, *agglomerative* clustering [61] with an *average linkage* parameter was used. Agglomerative clustering applies a bottom-up approach that attempts to merge similar clusters that do not exceed a maximum distance threshold. The maximum distance threshold was chosen by manually examining a few photographs in the dataset. It should be noted that determining the distance threshold requires knowledge about the target domain. If this is unfeasible, one can instead pre-determine the number of clusters by applying an objective measure of clustering quality. However, this approach may result in a number of clusters that does not allow meaningful conclusions to be drawn about the points of interest, for example if only two clusters are generated. A total of 81 clusters were generated by the clustering from which the four major points of interest were identified. It is important to note that some clusters correlated to different viewpoints of the same POI and were thus consolidated into one. The photographs contained within each cluster were then labelled according to the respective POI.

A clustering of the dataset of photographs showed four unique points of interest: the Depot as seen from the outside, the staircase including the atrium, the art storage, and the rooftop terrace. A portion of the photographs could not be clearly attributed as belonging to a certain point of interest and added into a separate miscellaneous category (’Misc’).

### Textual analysis by topic modelling and sentiment analysis

Two lead questions guided the textual analysis. The first, question is which topics users are talking about when they mention the Depot. The second question is, what criticism and praise users have for the building. The captions were pre-processed by translating them into English, lowercasing, lemmatising and removing non-meaning-bearing words, so-called *stopwords*, for example, the, and, etcetera. Additionally, the post description was separated from the trailing hashtags, which together constitute the post caption. To address the first lead question, a topic model was applied. *Topic modelling* is a method in natural language processing used to extract distinct topics from a corpus of texts by identifying words that tend to appear in the same context. Thus, a topic is characterised by a set of keywords most relevant to that topic. The topic modelling was conducted using Python and BERTopic [62]. Although BERTopic has been shown to outperform other topic modeling approaches, the author notes shortcomings in interpretability of the topics, especially when providing few text documents to the model. Therefore, how users are talking about the Depot was also explored by counting the occurrence of lemmas [63], specifically of adjective-noun combinations to capture user’s attitudes towards the Depot and discussed topics. This task required identifying adjectives in the post descriptions and relating them to a corresponding noun. Humans can perform this task effortlessly when reading a text, while in natural language processing it must be emulated through algorithms for part-of-speech tagging and dependency parsing. Take for example the sentence fragment ‘The (*determiner*) beautiful (*adjective*) Depot (*noun*)’, where the part of speech has been annotated in brackets. The sentence includes a dependency between *the* and *Depot* as well as *beautiful* and *Depot*. This dependency forms a tree-like structure with *depot* as the root and *the* and *beautiful* as children. *Beautiful* and *Depot* are then extracted as they form an adjective-noun pair. Transitive dependencies like in ‘I visited the Depot, and it was beautiful’, where *it* refers to *Depot*, were considered. An algorithm for extracting such adjective-noun pairs can be found below. Spacy [64] was used for part-of-speech tagging and dependency parsing.

Algorithm for finding pairs of adjectives with their corresponding noun in a corpus of text.

1 function find_adjective_noun_pairs(texts):

**Input**: A list (corpus) of texts

**Output**: A list of all adjectives in the corpus paired with their dependent noun

2 adjnouns ← empty list

3 for each text in texts:

4 dependency_tree ← parse_dependency(text)

5 for each word in text:

6 if is_adjective(word):

7 head ← word

8 while not is_noun(head) and not dependency_tree.is_root(head):

9 head ← dependency_tree.get_parent(head)

10 end

11 if is_noun(head):

12 tuple ← array([word, head])

13 adjnouns.append(tuple)

14 end if

15 end if

16 end

17 end

18 return adjnouns

This analysis resulted in a list of adjective-noun combinations ordered by their frequency of appearance. Sentiment analysis was used to investigate the second lead question regarding user opinions. In sentiment analysis, each text is assigned a value between -1 (very negative) and +1 (very positive). Values around 0 indicate a neutral sentiment. The sentiment analysis was conducted using Python and Spacy [64]. Notwithstanding, research by Roberts, Resch [65] describes the challenges of accurate identification of emotion by automated sentiment analysis of text extracted from Twitter, despite it having significantly more text in the datasets.

The deliberate act of timing the research to follow the live unfolding of an exceptional architecture and during the particularities of Covid-19 has disadvantages. A limiting factor in the textual analysis, especially in the topic modelling, was the low number of posts created by certain user groups, including tourists, given the relatively short observation period. Topic modelling, as typical for big data processing methods, requires a large number of inputs to yield interpretable results. The user groups ‘Private tourism outlet’ (53 posts) as a private actor and ‘Affiliated enterprise’ (72 posts) and ‘Depot Boijmans designer’ (62 posts) as a public actor yielded very limited insight into the topics discussed by the targeted users. The overwhelmingly positive and neutral sentiment in this dataset is presented with caution.

## 5. Findings and discussion

### A small percentage of users with high interactions shape the image of the Depot

**Fig 5:**
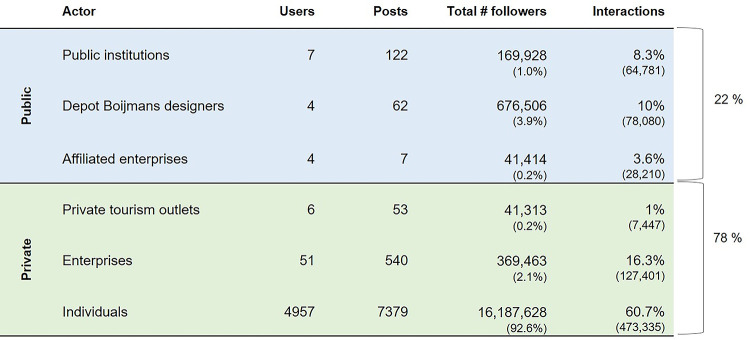
Actors and dataset interactions (by authors).

In the dataset, 20% (N = 1005) of users hold 83% (N = 647 000) of total interactions, these constitute likes and comments, across their posts. Therefore, the posts of a small group of users mainly shape the image of the Depot on Instagram. Considering the number of users in each category, total posts, total number of followers per actor, and percentage of interactions (*Fig 5*), it is worth noting that 78% of total interactions are from the private actors, while 22% are from the public actors.

The overall positive sentiment in captions is attributed to the private actors (78%, N = 7972) and not public actors (22%, N = 191) including the Depot’s designers (*Fig 5*). MVRDV posts (N = 62) are considered neutral and descriptive, for example "The depot is an experiment in adding nature to the city! It replaces the park space it takes up with an even larger public space above, with a 35-metre-high rooftop forest.⁠ The 75 birches, grasses, and 20 pines placed on the roof help retain water, promote biodiversity, and reduce heat stress in the city […]" [66], "The abracadabra doors of the depot Depot Boijmans Van Beuningen has eleven doors: three double and five single. When closed, some of these doors will seem to disappear entirely into the facade […]" [67]. This confirms that complimentary behaviour online mimics analogue behaviours, where an audience responds to an actor’s, or in this case, a building’s performance, rather than actors complimenting themselves as a means to generate positive interactions. Furthermore, the use of terms novelty and firstness are not seen as carrying a positive sentiment but rather as neutral since the Depot’s programme is in fact unique.

### Designers lead in generating high interaction posts

The highest percentage of interactions after the category ‘Individuals’ surprisingly emerges from private enterprises—these are predominantly photographers, media outlets and fashion outlets. Following behind are interactions with posts of the architects and designers of the Depot. This is important especially taking into consideration that the architects and designers category has the highest number of followers, with the MVRDV as the dominant actor, which has around 670 000 followers. Comparatively, the public institutions–which include a number of those who financed the project–account for 8.3% of interactions with 7 users. This demonstrates the weight of MVRDV as an actor and as a result the public actors play an active part in shaping content against which private actors could interact.

### Differences in posting behaviour of the various actors

**Fig 6:**
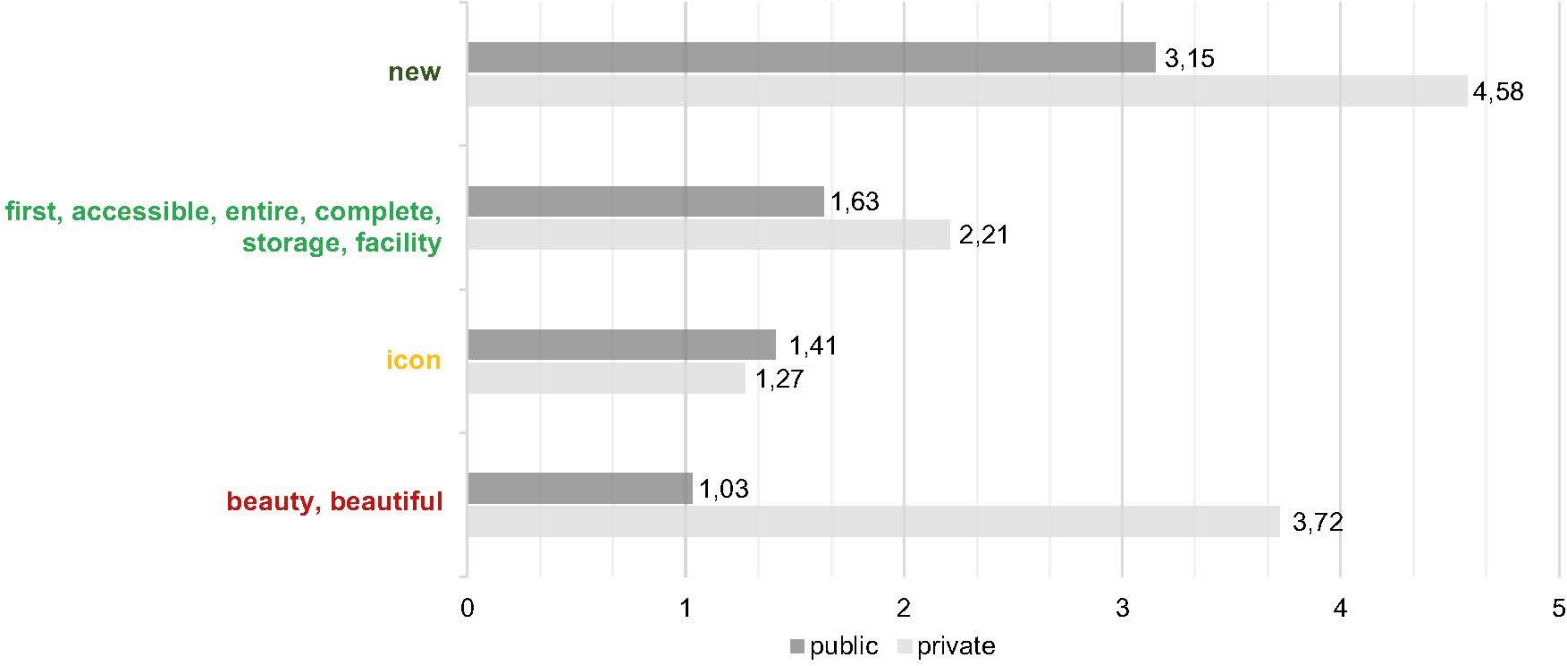
Length of descriptions (by authors).

All groups except the average user, ‘Individuals’, post longer descriptions (*Fig 6*). Overall, the average caption length between public (433.5±354.8 characters) and private posts (136.2±242.9 characters) showed large differences. A Mann-Whitney U test [68] revealed this difference to be significant (U = 1675660, N1 = 256, N2 = 7919, p<0.0001, one-sided). This indicates that while individuals post photographs to share a moment or an experience, the other user groups post descriptive texts that tell stories or alongside their photographs. Similar results have been reported by Egger et al. [31] who note that posts by official sources were more structured and planned than individual’s posts. Notwithstanding, this would need to be confirmed by manual analysis of the user’s posts after the topic modelling delivered very few topics which make the topic models of the different groups not comparable for most groups due to the low sample size, except individuals and enterprises. A qualitative investigation of posts of public actors to describe their narrative content was outside the scope of this research. As an example of manual analysis, a Chi-square test [69] of the number of image posted for each POI by the public and private actors in the time after the inauguration was conducted, similar to that by Acuti et al. [70]. The test showed a significance difference between images posted by the public and private actors (χ^2^ = 14.5, p = 0.002). Specifically, the outside view (public: 25.6%, private: 36.1% of photographs) and rooftop terrace (public: 23.2%, private: 10.9%) received different amounts of attention, while the art storage and staircase area were photographed in equal frequency (within 1% of each other). The over-representation of photographs of the Depot from the outside on the private actors is likely due to its façade being accessible in public space. Nevertheless, owing to the length of the posts and the emotional attributes, used it is possible to confirm that public actors communicate narratives. For example, the directors of the Museum Boijmans Van Beuningen Sjarel Ex and Ina Klaassen are quoted as saying, “We are convinced that making the collection accessible shows how much we care and how well we take care of it. This is something that the inhabitants of Rotterdam will be proud of; something that they want to see with their own eyes, because they partly own this enormous artistic treasure….” [71].

### Posting intensity is related to events not actors

**Fig 7:**
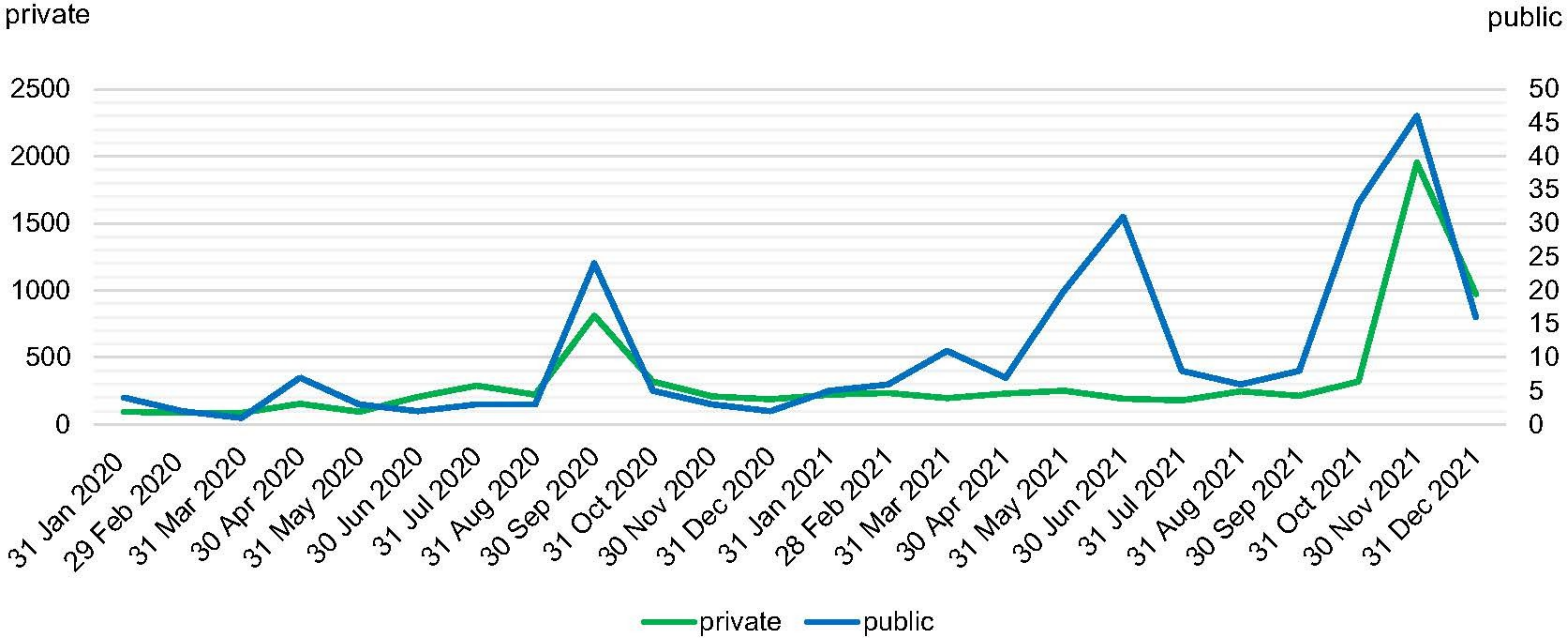
Longitudinal development of posts (by authors).

The actors posting behaviours show peaks at similar times coinciding with the events including the installation of the trees on the roof terrace in April 2020 and the four days of the silver opening in around 24 September 2020, for which reservation for 7000 places [72] was opened on the first day of September 2020 and was fully booked within one hour. Furthermore, peaks are related to the moving of the museum’s art collection starting in June 2021, a pop-up shop by Susan Bijl in June 2021, and the inauguration of the building on 6 November 2021 (*Fig 7*).

It is worth noting that that leading up to the major opening events, the public actors increase their posting activity a few days (before the silver opening) or even a few weeks (before the inauguration) before the private actors. The public actors peak in post activity in June 2021 demonstrates no significant change in post frequency as a response from the private actors. It is notable that across the time frame of the research the Covid-19 pandemic regulations limited access to the Depot. We conclude from this that the public actors’ posts do not stimulate private actors’ posts or vice versa. These peaks are related directly to each event with announcements of such events appearing on other webpages rather than in Instagram.

### Public actor and private actor topics converge

Natural Language Processing (NLP) was used to analyse the usage of adjective-noun combinations in captions, with which users describe the Depot. Fig 6 shows the in-caption frequency of the topics emerging from this analysis. The frequencies are normalised to 1000 characters to accommodate for differing average caption lengths between private and public actors.

Across all actor groups, when users post about the Depot they dominantly mention: *opening/newness* captured in phrases such as ‘new depot‘ and ‘new building‘, but what is novel about the programme is the concept of the building being the first publicly accessible art storage facility where the entire repository of artefacts are accessible captured in phrases such as ‘first depot‘, ‘accessible depot‘, ‘first facility‘, ‘entire collection‘, ‘complete collection‘ and ‘accessible facility’. Unique to the private actors is the use of the dominance of the term ‘beautiful‘ (*Fig 6*). The reference to the *opening* and *newness* is not surprising. Although, it is surprising that the topic of *firstness* is actively talked about by private actors to an extent that exceeds the activity by the public actors. Keeping in mind that *firstness* is difficult to communicate because the depot was still not open to the public at the time of this research, and it is visually difficult to communicate this through photographs—this can only be communicated via photographs of the art storage. For example, *firstness* would be captured in photographs of artworks and sculptures that are stored together because of their materiality and not curated as per conventional museum practice–that is according to painter or epoch. These indicate that both groups independently converged on topics about the Depot, i.e. they have separate but similar depictions about the Depot.

### Content is consistent over time with little influence from public actors

**Fig 8:**
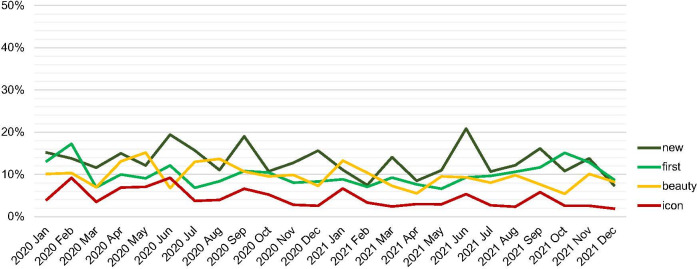
Development of terms as percentage of posts over time (by authors).

When comparing posts that contain the terms related to *newness*, *firstness*, *iconicity*, and *beauty* with posts that do not contain these terms, it is possible to identify a consistent proportion across the time frame (*Fig 8*). This indicates that content communicates stable topics over time.

**Fig 9:**
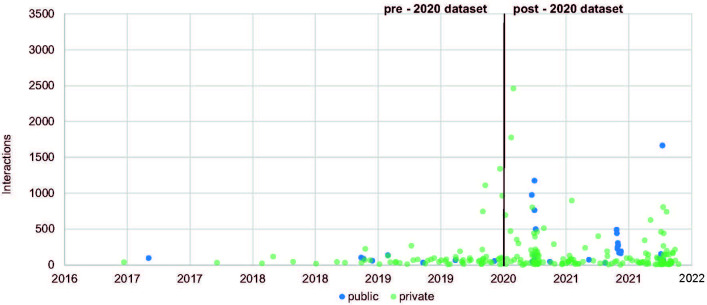
Occurrence of term ‘icon*’* in posts over time per actor group (by authors).

An investigation on how the usage of the terms evolved over time shows that there is little influence from public actors on private actors. The public actors’ posts with high interaction values are not followed by an increased number of private actors’ posts repeating the same term (*Fig 9* shows the case of the term ‘icon’). Owing to the timeframe of the research these topics might be skewed and it is indeed expected that with the passing of time, the percentage of posts about "new" (building) for example, will decline, in favour of posts about other subjects, but references to the uniqueness of Depot’s programme may not decline. While the discourse may change after the inauguration, the frequency of these topics does not increase in the time before the inauguration. This has implications for the propagation of narratives from public to private on Instagram.

### Both the exterior and the interior atrium of the Depot are potentially equally iconic

**Fig 10:**
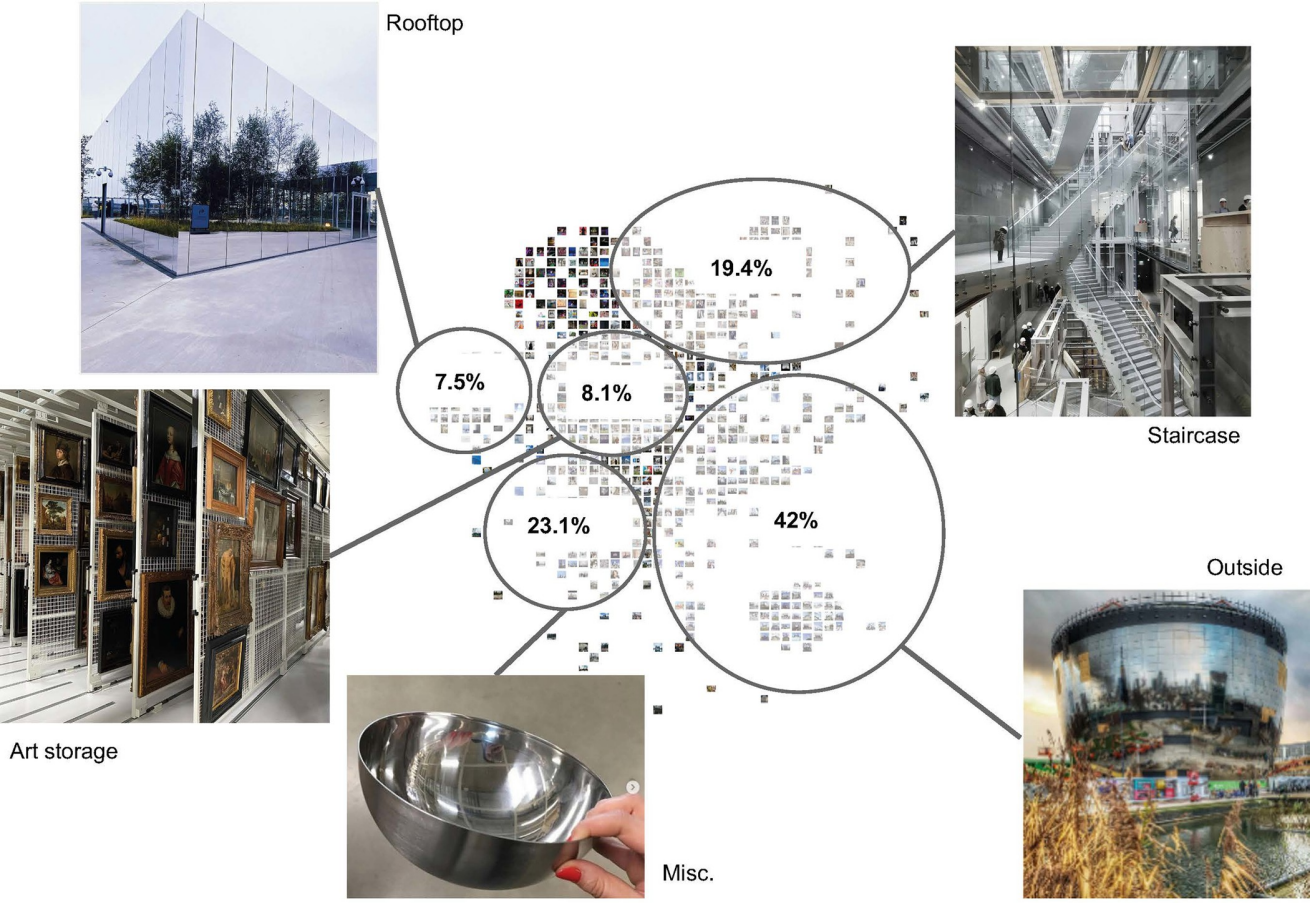
Visualisation of the image clustering (by authors).

The content of photographs offers further understanding of what visitors find worth communicating about the Depot. Four points of interest emerged from the image clustering and labelling. These are as follows: exterior photographs of the building (both day and night time photographs, and during construction) at 42%, the atrium with the staircase at 19.4%, the rooftop at 7.5%, art storage at 8,1% and a miscellaneous cluster at 23,1%. The miscellaneous cluster includes schematic drawings of the building, and interestingly also the silver Ikea salad bowl which is alleged to have inspired the form of the final building (*Fig 10*). The exhibition pieces themselves receive only marginal interest from visitors. Similar observations have been made by Rhee et al. [57], where the clustering of photographs revealed that the museum’s architectural features are more frequently photographed than the art itself in certain museums.

Not surprisingly the Depot is photographed from the exterior predominantly by passers-by whilst the building is closed and during its construction phase. General public access took effect on 6 November 2021 and then was still subject at time to limitations by entry fees and lockdown regulations in the study period. This changed the distribution of photographs dramatically.

**Fig 11:**
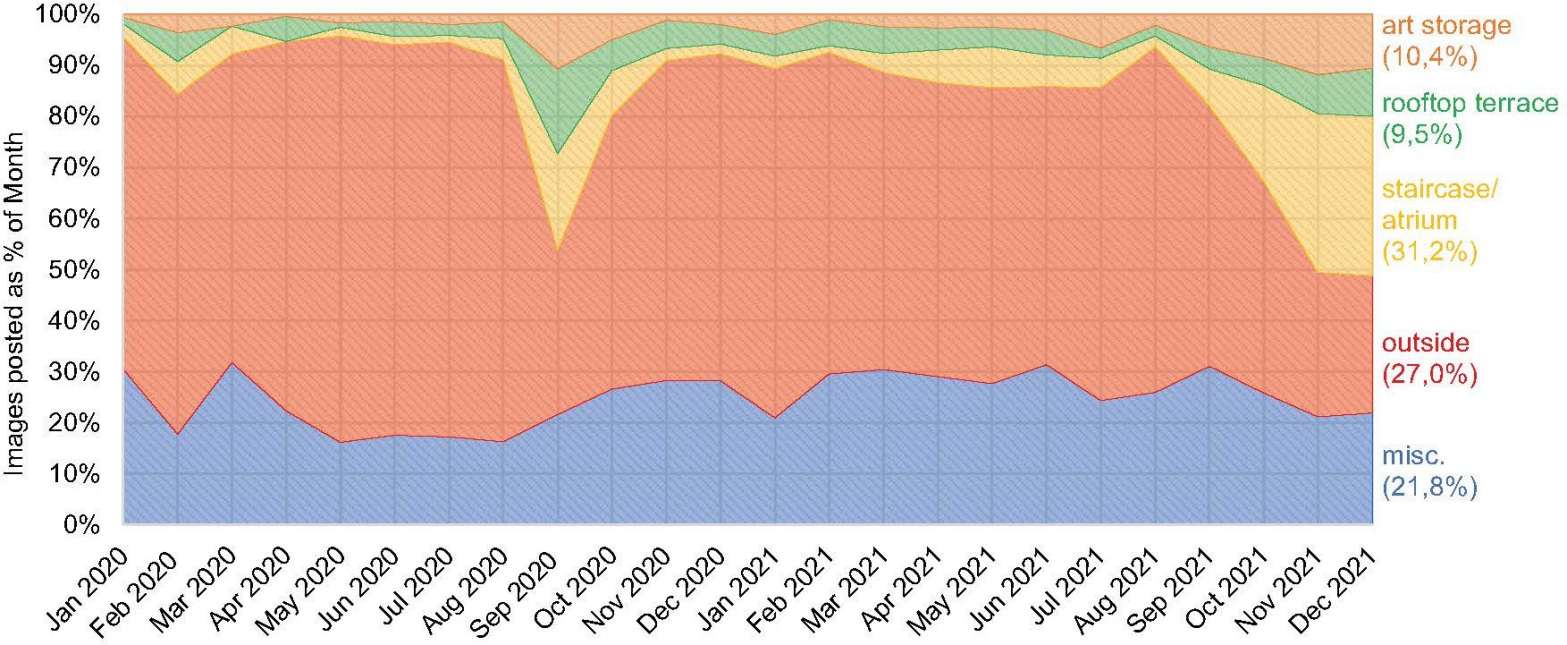
Photograph frequency by point of interest (by authors).

The analysis of how the distribution of photographs in the clusters evolved over time makes obvious pre- and post-inauguration periods (*Fig 11*). When the building became accessible to the public, the percentage of photographs of the atrium and staircase and the exterior has become more similar as demonstrated by 31.2% for the former and 27% for the latter. It is important to note that since the opening it is possible to enter the atrium and take photographs of the staircase without paying an admission fee, and gain access to the rooftop café restaurant which was not operational at any time during the period of research. Therefore, it is expected that access to building will play less of a role in the future distribution of photographs as artist installations on the exterior at night may be offset by paid visits to the art storage in opening hours. With its opening, the staircase of the Depot was revealed alongside the exterior as potentially equally iconic.

### Sentiment analysis findings can be presented only with caution due to the constraints of Instagram as a platform

Instagram has limited text and it was selected because of its capabilities to communicate the visual nature of architecture and its interaction functionalities. The findings are presented with caution as the credibility of automated sentiment analysis in the field of urban planning is questioned and manual sentiment is considered more robust [65, 73]. The sentiment analysis of the captions revealed that there was an overall neutral to positive sentiment in the post captions (average sentiment score: 0.14).

**Fig 12:**
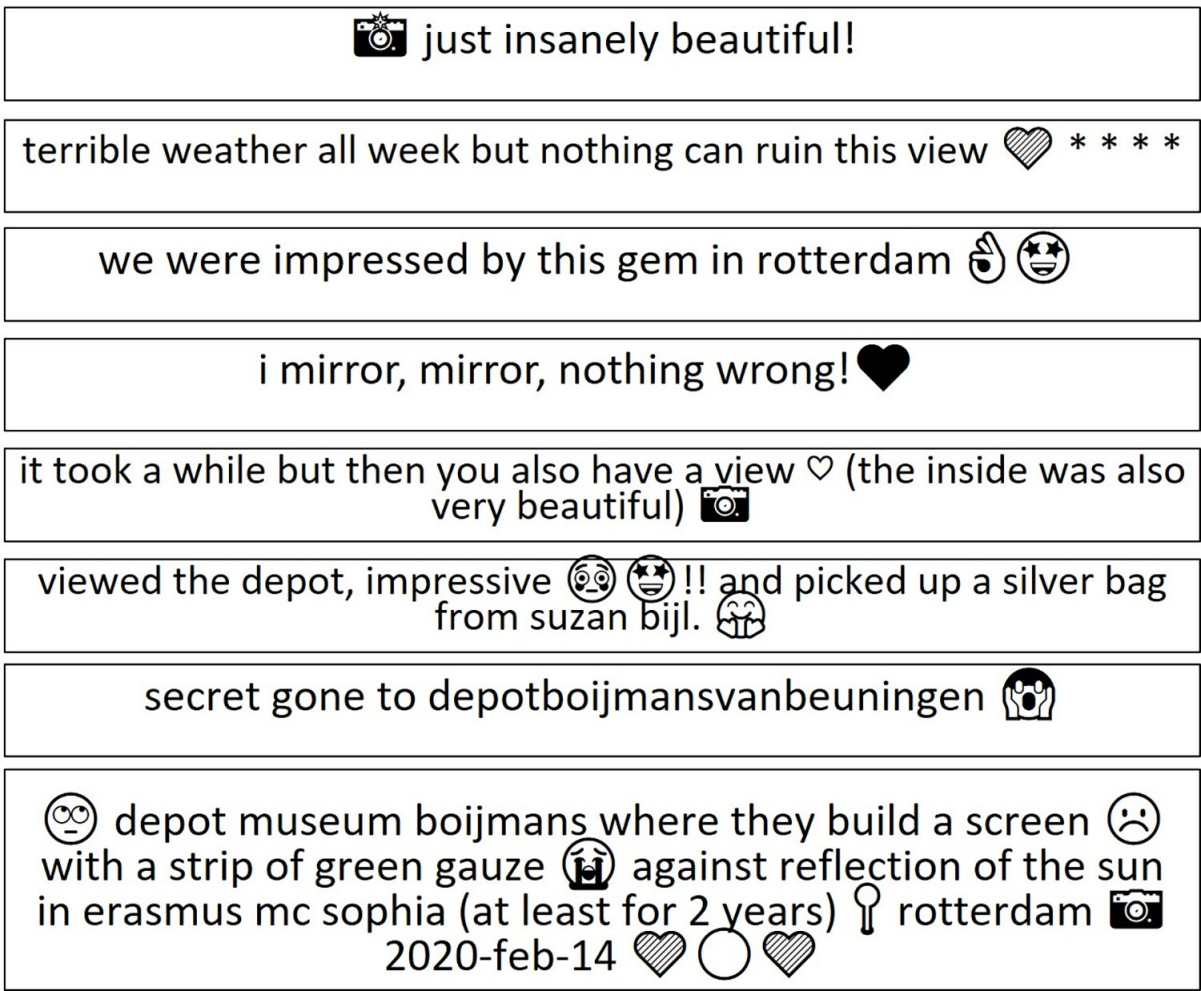
A selection of comments with emojis (by authors).

The analysis of the results suggest that very positive captions typically focused on the visual aspects of the Depot. Fig 12 includes a few exemplary comments. A manual review of the captions labelled as expressing a negative sentiment revealed only a fraction of these comments are directed at the Depot itself. In fact, negative sentiments were typically related to the weather (‘a cold sunday afternoon….’) or the result of a misclassification of comments where the sentiment could be interpreted in multiple ways (‘[…] you can take a lot of crazy photos in boijmans’ mirroring […]’. Furthermore, words such as ‘insanely’, ‘crazy’ or for example ‘wrong’ in the caption have a negative connotation when they stand alone, but in the phrase, with an emoji, in relation to the photograph, and taking into consideration the use of colloquial language, these carry positive sentiment. Despite the sentiment analysis results being valid it cannot identify complexity or abstraction in captions but is successful in categorising based on shallow level, that is whether users liked or disliked their trip to the depot. The limited number of negative comments about the Depot asks for further research into the perceptions of an “Instagram positivity bubble” mentioned earlier in this paper.

## 6. Conclusion

This research offers methods and their applicability toward a catalogue for data analysis for architecture and urban studies. The authors’ previous research [73] addressed the locational aspects of hash tagged photographs in Flickr, research findings in this paper address the aspect of actors, their post behaviours and influence on Instagram. As such, findings indicate that public and private actors differ in their post behaviour in timing and nature of the text, with public actors posting longer stories and communicating narratives. Public and private actors post about similar topics and in the same frequency. This leads to the conclusion of converging but independent depictions of the Depot between public and private actors. The convergence of content generated from the public and private actors on Instagram and thus the co-creation of the building’s image has been observed in tourism studies [22, 31]. The findings offer the perspective that while the content posted by both public and private actors aligns, this may not be the result of content being projected and received on Instagram. Rather, it is conceivable that the respective narratives have been exchanged on other (social) media or that such a convergence happened entirely independently.

It is especially notable that private actors talk about topics such as iconicity and the novelty of the art storage facility, which are topics that one would attribute to public actors. Although in his speech at the silver opening event [74], Winny Maas, cofounder of MVRDV, spoke of the negative connotation of the term ‘icon’, which suggests the topic of iconicity will not have emerged from the designers. It may be argued that private actors are talking about these topics because there is an overrepresentation of locals due to the pandemic and locals, as opposed to tourists, and the former care a lot about topics such as iconicity or the functionality of a newly built cultural amenity in their home city. The topics of iconicity and novelty over the whole observation span have a constant frequency implying the discourse has been established on both public and private actors and is not evolving. This is particularly evident in that posts from public actors do not lead to more post activity from private actors in the days or weeks following the public post. Neither is there a stickiness of posts but this may have to do with the lack of functionality for sharing and resharing posts in Instagram. Together these findings provide weak evidence for this convergence being the result of one of the actors pushing a narrative, and stronger evidence for convergence happening independently. What is evident is that Instagram is not a magical place where narratives are created by certain actors and automatically received and reflected back by others. Whether this is this due to the nature of the platform and its focus on photographs, or that the comments are not immediately visible on feeds remains a topic of future research.

The potential effect of the Depot on the existing Museum Boijmans van Beuningen as a signpost or spectacular representation of itself may be similar to the IM Pei pyramid at the Louvre Museum in Paris. Given the ordinariness of the older building of the museum and the subdued character of the pending Mecanoo extension, the museum will probably benefit from the Depot’s signposting power. However, the Depot’s assimilation to become an icon in the Museum Park or even the City of Rotterdam can obviously not be tested at this early stage of its lifespan. The lasting effects related to the circulation of interior and exterior photographs cannot be asserted. The argument that iconicity develops over time through depth of meaning, symbolism and the appropriation of the building by the public may become evident in photographs including selfies, memes, in nicknames for the building and associated hashtags. What is most interesting for future research is the extent to which social media contributes to the speed at which buildings transform into icons.

The social media platform acts as a piece in a puzzle that contributes towards the spreading, amplification and perhaps also making of narratives that support a building’s acquisition of iconic status. If the user generated content is, at least for this case of the Depot, autonomous then that which influences the private actor’s content beyond the public generated content remains to be uncovered. Here, qualitative analysis of the photographs might provide a clue, because individuals post shorter captions and what is captured and communicated via the photograph is possibly more important than the text. Photographs capture complex stories, and as the role of photographs changes from that of documentation to being a communication tool, so too does the agency of the photograph. Beside the actors, the photograph itself becomes an actor suggesting the data has a certain power and in the “widest sense may affect and be active in cultural and social situations and events” [75].

At this point we are confronted with a number of avenues for future research to unlock the power or agency of the photographs by uncovering the narratives that the photographs are communicating including what is communicated through selfies and memes of exceptional architecture. As such, research into the development of automated methods to analyse of photographs themselves [76] is pertinent for the urban development field. In addition promising directions for research include the study of emojis which is considered intrinsic to the understanding of sentiment in posts [77]. Besides the sentiment of social media platforms themselves research into the positive and negative impacts of using platforms is required. Furthermore, it is worthwhile to investigate how negative sentiment proliferated can have consequences such as that of “The (Marble Arch) Mound” also by MVRDV, a temporary landscape installation [78] that was removed earlier than planned after intense criticism in the media.

Beyond methods and direction of future research, our research to date demonstrates evidence for measuring popularity of exceptional architecture using social media posts, but not the making of iconicity. The Depot shifts the focus from the art itself to the institutional management of art storage as an art using exceptional architecture. The design intention to clad the building in mirrored glass to reflect not only the city but the way people use and interact with the facade contributes to the “instagrammability” of the architecture. This raises a broader question of how architectural design practice is affected by the pressure to reap the short-lived benefits of performing on the social media, especially if the assumption is that popularity paves the way to iconicity.

## Supporting information

S1 Data Depot Boijmans data. (CSV)
